# Hand Rehabilitation Robotics on Poststroke Motor Recovery

**DOI:** 10.1155/2017/3908135

**Published:** 2017-11-02

**Authors:** Zan Yue, Xue Zhang, Jing Wang

**Affiliations:** School of Mechanical Engineering, Xi'an Jiaotong University, Xi'an 710049, China

## Abstract

The recovery of hand function is one of the most challenging topics in stroke rehabilitation. Although the robot-assisted therapy has got some good results in the latest decades, the development of hand rehabilitation robotics is left behind. Existing reviews of hand rehabilitation robotics focus either on the mechanical design on designers' view or on the training paradigms on the clinicians' view, while these two parts are interconnected and both important for designers and clinicians. In this review, we explore the current literature surrounding hand rehabilitation robots, to help designers make better choices among varied components and thus promoting the application of hand rehabilitation robots. An overview of hand rehabilitation robotics is provided in this paper firstly, to give a general view of the relationship between subjects, rehabilitation theories, hand rehabilitation robots, and its evaluation. Secondly, the state of the art hand rehabilitation robotics is introduced in detail according to the classification of the hardware system and the training paradigm. As a result, the discussion gives available arguments behind the classification and comprehensive overview of hand rehabilitation robotics.

## 1. Background

Stroke, caused by death of brain cells as a result of blockage of a blood vessel supplying the brain (ischemic stroke) or bleeding into or around the brain (hemorrhagic stroke), is a serious medical emergency [1]. Stroke can result in death or substantial neural damage and is a principal contributor to long-term disabilities [1, 2]. According to the World Health Organization estimates, 15 million people suffer stroke worldwide each year [3]. Although technology advances in health care, the incidence of stroke is expected to rise over the next decades [4]. The expense on both caring and rehabilitation is enormous which reaches $34 billion per year in the US [5]. More than half of stroke survivors experience some level of lasting hemiparesis or hemiplegia resulting from the damage to neural tissues. These patients are not able to perform daily activities independently and thus have to rely on human assistance for basic activities of daily living (ADL) like feeding, self-care, and mobility [6].

The human hands are very complex and versatile. Researches show that the relationship between the distal upper limb (i.e., hand) function and the ability to perform ADL is stronger than the other limbs [7–9]. The deficit in hand function would seriously impact the quality of patients' life, which means more demand is needed on the hand motor recovery. However, although most patients get reasonable motor recovery of proximal upper extremity according to relevant research findings, recovery at distal upper extremity has been limited due to low effectivity [10]. There are two main reasons for challenges facing the recovery of the hand. First, in movement, the hand has more than 20 degree of freedom (DOF) which makes it flexible, thus being difficult for therapist or training devices to meet the needs of satiety and varied movements [11]. Second, in function, the area of cortex in correspondence with the hand is much larger than the other motor cortex, which means a considerable amount of flexibility in generating a variety of hand postures and in the control of the individual joints of the hand. However, to date, most researches have focused on the contrary, lacking of individuation in finger movements [12, 13]. Better rehabilitation therapies are desperately needed.

Robot-assisted therapy for poststroke rehabilitation is a new kind of physical therapy, through which patients practice their paretic limb by resorting to or resisting the force offered by the robots [14]. For example, the MIT-Manus robot uses the massed training approach by practicing reaching movements to train the upper limbs [15]; the Mirror Image Movement Enabler (MIME) uses the bilateral training approach to train the paretic limb while reducing abnormal synergies [16]. Robot-assisted therapy has been greatly developed over the past three decades with the advances in robotic technology such as the exoskeleton and bioengineering, which has become a significant supplement to traditional physical therapy [17, 18]. For example, compared with the therapist exhausted in training patients with manual labor, the hand exoskeleton designed by Wege et al. can move the fingers of patients dexterously and repeatedly [19, 20]. Besides, some robots can also be controlled by a patient's own intention extracted from biosignals such as electromyography (EMG) and electroencephalograph (EEG) signals [21, 22]. These make it possible to form a closed-loop rehabilitation system with the robotic technology, which cannot be achieved by any conventional rehabilitation therapy [23].

Existing reviews of hand rehabilitation robotics on poststroke motor recovery are insufficient, for most studies research on the application of robot-assisted therapy on other limbs instead of the hand [23]. Furthermore, current reviews focus on either the hardware design of the robots or the application of specific training paradigms [23, 24], while both of them are indispensable to an efficient hand rehabilitation robot. The hardware system makes the foundation of the robots' function, while the training paradigm serves as the real functional parts in the motor recovery that decides the effect of rehabilitation training. These two parts are closely related to each other.

This paper focuses on the application of robot-assisted therapy on hand rehabilitation, giving an overview of hand rehabilitation robotics from the hardware systems to the training paradigms in current designs, for a comprehensive understanding is pretty meaningful to the development of an effective rehabilitation robotic system. The second section provides a general view of the robots in the entire rehabilitation robotic system. Then, the third section sums up and classifies hardware systems and the training paradigms in several crucial aspects on the author's view. Last, the state of the art hand rehabilitation robotics is discussed and possible direction of future robotics in hand rehabilitation is predicted.

## 2. An Overview of Hand Rehabilitation Robotics

### 2.1. Overview

Robot-assisted poststroke therapy for the upper extremity dates back to the 1990s [17]. It has been greatly developed over the past decades with the progress in robotic technology and has been a significant supplement to traditional physical therapy. The hand rehabilitation robots were first designed to do heavy labor work as a better replacement for human therapists. These early robots focus on the design of structure, actuator, method of control, and so forth to achieve a robotic hand that is more adaptive to the motion characteristics of bones and joints and meets the needs of rehabilitation more effectively [25].

**Figure 1:**
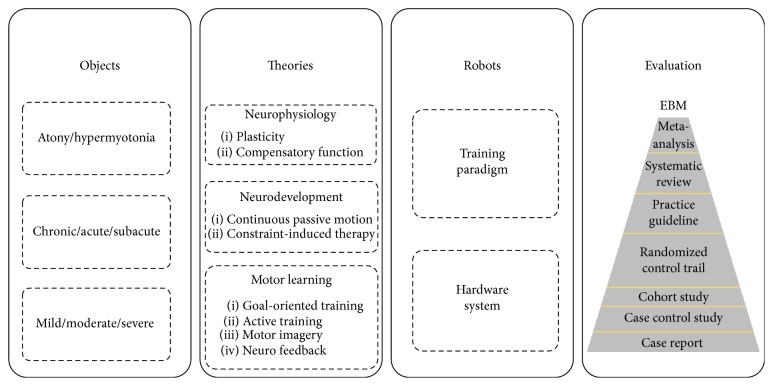
An overview of the hand rehabilitation robotics.

Now, the hand rehabilitation robotics has been greatly developed with the rapid development of neurosciences and clinic knowledge, which makes the design of hand rehabilitation robotics for poststroke rehabilitation become more complex for involving multidisciplinary knowledge such as anatomy, neurosciences, cognitive and learning science, and rich experience in the clinic [23, 26–28]. Apart from the robots themselves, knowledge of stroke patients' differences, rehabilitation theories, and the evaluation are all essential for the design of an effective hand rehabilitation robot. Here, a summary of this core content of hand rehabilitation robotics in rehabilitation is made to give the researchers an overview of hand rehabilitation robotics. The contents are displayed in Figure 1.

#### 2.1.1. Subject Differences

Motor impairment of stroke patients differs from person to person, and states and conditions vary in the course of recovery. Factors such as types of muscle state (e.g., atony or hypermyotonia), phases of stroke (chronic, acute, or subacute), and levels of stroke (from mild to severe stroke) should all be taken into consideration for individualized treatment [29]. For example, patients with atony cannot use the assistive robots that require residual motion ability or too many sets of force training which may cause the abnormal motion modalities. The benefits of using the hand rehabilitation robotics are that the robotic technology can be used to quantify and track motor behaviors or biosignals for individual patients, thus the rehabilitation robotics can represent the sophisticated method existing today for precisely driving therapeutic engagement and measuring precise outcomes [30]. Lacking consideration of the difference between subjects may greatly decrease the efficacy of robot-assisted therapy.

#### 2.1.2. Theories for Rehabilitation

Although current theories underlying the motor recovery are insufficient, a few of existing rehabilitation theories are still instructive and significant for the application of robot-assisted therapy. Three main popular directions are the theories of neurophysiology, neurodevelopment, and motor learning. The neurophysiology mechanism, such as the plasticity and compensatory function, is the theoretical basis of the poststroke motor recovery [14]. The neurodevelopmental treatment (NDT), or the Bobath concept, which mostly formed from clinical experience, focuses on normalizing muscle tone and movement patterns in order to improve recovery of the hemiparetic side and inspire training strategies such as continuous passive motion [31] and constraint-induced therapy [14, 32]. The learning theories, such as the Hebbian learning or motor relearning which instructed many strategies including the goal-oriented training, active training with all kinds of feedbacks, are the current trend in hand rehabilitation robotics [33–37]. Although there is not much specific convincing evidence for these strategies, many applications in cooperation with the robotic hand have been practically used in rehabilitation and have achieved some good results. Deeper researches on the better application of these rehabilitation theories would be the foundation of future design of RHH.

#### 2.1.3. Application of Hand Rehabilitation Robotics

The application of hand rehabilitation robotics is to achieve effective rehabilitation by making a serial of decisions on the designing of both hardware system and the training paradigm [14, 23, 26]. For designs that focus on the hardware system, they solve problems such as the safety issues of mechanism and the portability or flexibility of devices. For example, Wege et al. adopt an across joint linkage structure to solve the joint coordination problem [19, 20], while a finger can be flexibly controlled with four degrees of freedom. In et al. design an underactuation jointless exoskeleton that can be safe in motion and light in weight [38]. For designs that focus on the paradigm, they deal with the interaction between human and robot to promote motor relearning. For example, Ueki et al. and Sarakoglou et al. develop a system that provides the feedback of real-time state in virtual reality (VR) scene, to enrich the perception of motion in training [39, 40]; Sarakoglou et al., Hu et al., and Ramos et al. study the hand rehabilitation robot, respectively, controlled by patients' own force signal, EMG signal, and EEG signal, to generate patient participation in training [40–42]. For a hand rehabilitation robot system, hardware system and the training paradigm are both essential and dependent to each other. Detailed classification and introduction about these two parts in the application of hand rehabilitation robots would be discussed in the next section.

#### 2.1.4. Evaluation of Treatments

The evaluation of rehabilitation robotics reflects the recovery effects of human functions, which make it imperative for a complete design. There are varied ways on evaluating the robots in current researches, including the performance on improving range of motion (ROM) of joints, the velocity of motion, the force exerting to hands, and the executing of functional tasks. A set of classic clinical scales is also used to evaluate the design by measuring the recovery of stroke patients. The most frequently used scales include the Arm Research Assessment Test (ARAT), Fugl-Meyer Assessment (FMA), and Motor Activity Log (MAL) [43]. It should be focused on the evaluation of rehabilitation robotics that is different from traditional robots, because of the special clinical applications. To the validation before clinical use, a therapy has to follow the principle of evidence-based medicine (EBM), which indicates that many results of current research are collected to demonstrate the usefulness [44]. For example, a surprising finding from the results of independent researches shows the usefulness of current hand rehabilitation robots while a research under the principle of EBM gives weak demonstration for the usefulness [45]. It might be explained by the small sample sizes and low quality of the experiment that limits the supportive evidence in the current research of hand rehabilitation robotics [46]. That shows the reason why the validation of robots, especially the designing of more standard randomized controlled trials (RCTs) with large-scale samples and high quality, is so imperative for rehabilitation robotics.

### 2.2. Requirements

The requirement of hand rehabilitation robotics is not the same as the traditional design of robots, because it involves humans. Some special matters like medical principles and applications in clinic need to be paid attention to, for example, the safety and the availability of hand rehabilitation robots. Although some other requirements such as participation are shown to be meaningful, these may not be essential for every kind of robot-assisted therapy. Two of the most imperative requirements are introduced here.

#### 2.2.1. Safety

With the participation of human, safety becomes the top priority for the design of rehabilitation robotic hands. Any mechanical problems would do serious harm to the human bodies because of the close contact between the robotic hand and the human hands [25]. Therefore, enough considerations for the motion characteristics of fingers are imperative in the design. For instance, the center of rotation of linkage structure should coincide with the rotational axis of the human joint or the mechanical stopper should be used for setting limits to the range of motion [47]. These mean that the hand anatomy and potential emergency have to be anticipated in the design of any robotic exoskeletons by the designers [26, 48, 49].

#### 2.2.2. Availability

The motor recovery training is long term and costly to most patients. Some researches show that the main limitation to the practical use of hand rehabilitation robotics is the availability [50]. The availability is decided by the expense of the device and the complexity of an operator [23]. For instance, complex rehabilitation robotic devices require supervision from specially equipped therapists with extra wage expense [50]. Most of current hand rehabilitation robots offer 1 degree of freedom (DOF) for a single finger while fingers have far more DOF, since any extra DOF would bring considerable extra expense [51]. Big equipment for rehabilitation is very expensive, and the burden is too heavy for the hospitals to provide enough room and equipment for stroke patients [52, 53]. The growth of stroke population and the condition of current rehabilitation indicate that in-home treatment would be the trend of hand rehabilitation robotics. The transformation of application from the lab to the home is in urgent need of improving in availability of hand rehabilitation robotics.

## 3. Classification of Hand Rehabilitation Robots

**Figure 2:**
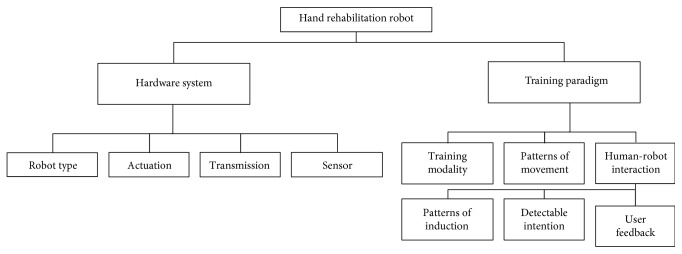
The classification of hand rehabilitation robot.

There exist many kinds of ways for the classification of hand rehabilitation robots, some of which follow the convention in mechanical design (focus on the hardware system), while others follow the convention in rehabilitation (which focus on the training paradigms) [23, 26]. In fact, each of these ways of classification has its own value, and they are dependent to each other. For example, the hardware system depends on the basic abilities of the rehabilitation robotics (e.g., possible movements and feedback information), while the training paradigms are the main functional components in the recovery (e.g., the application of specific rehabilitation theories). To date, papers reviewing the hardware system and training paradigms have been separated to each other or just from one special angle [14]. Here, both of them are overall reviewed in the next subsection. Notably, this paper will not give the most specific details about the classification of rehabilitation, but tries its best to give an overview of the position of each component and the relationship between them. The overall overview in this classification is shown in Figure 2.

### 3.1. Hardware System

The hardware system is the foundation of hand rehabilitation robots. It decides the possible types of motion the robots can offer to the patients and the possible signals the robots can obtain from patients. The hardware system can be classified in details in a mechanical classification [26]. However, here, the classification is made in cruder categorization for highlighting several most important aspects in the design of hand rehabilitation robots. The hardware systems are divided into aspects including types of robots, the actuations, types of transmission, and sensors. Other aspects such as power are not mentioned because they are not the critical parts for the hand rehabilitation robots, and details about these have already been discussed by other researchers [24].

### 3.2. Robot Type

**Figure 3:**
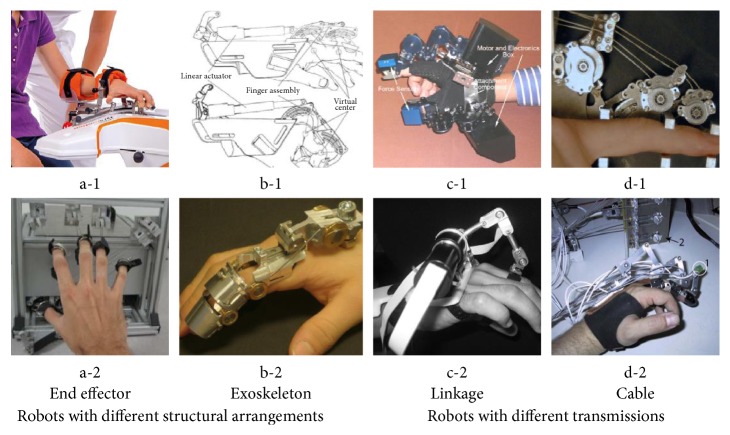
Examples of different kinds of robots [10, 19, 20, 54, 55, 57, 58, 74, 76–78].

Existing hand rehabilitation robots can be divided into two main types according to the alignment of the device and the user: the end effector and the exoskeleton. The end effector is external to the patients' body while the exoskeleton is worn by human beings (Figure 3).

#### 3.2.1. End Effector

The end effector is external to the body of patients, and it provides required force to the end of the user's extremity to help or resist the motion [26]. For example, the AMADEO robotic system (Figure 3, a-1) designed by an Austrian is already a commercial product. After fastening, the finger supports the finger tips and thumb and bending and stretching movements can be performed followed the slider [54]. The HandCARE (Figure 3, a-2) is another end effector designed by Dovat et al., in which each finger is attached to an instrumented cable loop allowing force control and a predominantly linear displacement [55]. The end effector provides force without considering the individual joint motions of the patients' limbs, which bring problems such as the limited range of motion and the dead point issues [56]. Furthermore, the end effector is not portable for being external to the human body, which limited the practical use in clinic.

#### 3.2.2. Exoskeleton

Different from the end effector, exoskeleton device can be worn on the body of patients. The joint and links of the robot have direct correspondence with the human joints and limbs, respectively [26]. For example, Ho et al. develop a wearable hand rehabilitation robot that offers 2 DOFs for each finger (Figure 3, b-1) [10, 57]; Chiri et al. designed the HANDEXOS which is low in overall size and light weight (Figure 3, b-2) [58]. This portability of exoskeleton makes it a good choice for stroke rehabilitation, especially for patients in the later period of stroke when they can train themselves at home [24]. Although there are problems such as the robot axes have to be aligned with the anatomical axes of the hand, the exoskeleton robots are widely used in the rehabilitation robotics and have been greatly developed these years. The mention of functional degrees of freedom (fDOF) which offers a method for using less complex actuation strategies to simplify the complex multi-DOF movements [24, 59] and the development of soft-bodied robots [60] both promote the application of exoskeleton robotics. In et al. designed the underactuated jointless robots that are very light in weight [38]. Now, the exoskeleton robots have been the trend of hand rehabilitation robots in poststroke rehabilitation.

### 3.3. Actuation

The function of actuation is to transform different kinds of energy to actuate the motion of robots. There are 5 kinds of actuation mentioned here: elector motor, hydraulic, pneumatic, pneumatic muscle, and human muscle. Although there are still some other kinds of actuation such as the piezoelectric and the shape memory alloy which are promising for being thin and lightweight, they are not mentioned here for either being limited by their own technical dilemma in practical application or just being theoretical design [61–64].

#### 3.3.1. Electrical Motor

The electrical motor is almost the most widely used actuation in design of hand rehabilitation robots, because they are easily available, reliable, and easy to control and with high precision. For example, the HANDEXOS designed by Chiri A et al. is actuated by the force transmitted for DC motor to a Bowden cables; the exoskeleton hand robotic training device designed by Ho et al. is actuated by micro linear electrical motor [10, 58]. The general performance in the torque-velocity space makes the electrical motor useful in applications such as hand rehabilitation robots where variability in control strategies is sought-after [24]. The might disadvantage of the electrical motor is that the rigid structure of electrical motor might bring safety problems. Nevertheless, the electrical motor can be controlled in torque, which makes the actuator able to get the information of the robots without the need of extra sensors.

#### 3.3.2. Pneumatic

The pneumatic actuators are used much less than the electrical motor in hand rehabilitation robots, such as the ASSIST designed by Sasaki et al. This actuator has advantages such as less requirement of maintenance and can be stopped under a load without causing damages [26, 65]. Although problems like noise can be overcome by using precompressed air storage, the problem of size cannot be settled because the air storage chamber is necessary. Thus, the pneumatic actuator might better be used for systems with lower mobility.

The development of pneumatic artificial muscle makes the actuation another choice. The pneumatic muscle made of rubber inner tube with a shell can inflate or contract. For example, the commercial hand rehabilitation robotic system produced by Kinetic Muscles Inc. (USA) is actuated by air muscle actuator [66]. There is also another kind of pneumatic muscle, namely, the bending type pneumatic muscle. For example, the pneumatic rubber muscle was designed by Noritsugu et al. [67]. The disadvantage of pneumatic actuation is that the actuators are difficult to control for its time variability and nonlinear.

#### 3.3.3. Hydraulic

The hydraulic actuators are very good in performance such as can generate higher torque compared with the electric or pneumatic systems and can be controlled in high precision and frequency [68]. But, the requirement of a wider space to accommodate the oil transmitting pipes and conduits makes the hydraulic actuators seldomly used in hand rehabilitation robots [26].

#### 3.3.4. Contralateral Extremity

The contralateral extremity can be thought as an actuation too. The hand rehabilitation robots actuated by the bilateral limb are usually used in the robotic system applying the bilateral training strategies [69]. The robots for the impaired hand can be directly actuated by the force offered by the healthy extremity or indirectly actuated in a synchronized control by the signal obtained from the healthy hand [70, 71]. To date, only the latter one has been researched. For example, Rahman et al. designed the bilateral therapeutic hand device, in which the exoskeleton was worn on the impaired moves according to the data from the glove worn on the healthy hand [71].

#### 3.3.5. Human Muscle

The human muscle on the impaired hand can be activated by functional electrical stimulation (FES) to complete the motion of impaired hand the same as robotic actuation [72]. Moreover, some applications combine it with the robotic actuator for rehabilitation. Thus, the human muscle can be classified as a kind of actuation in a broad sense here. Rong et al. have proposed an FES & robotic glove rehabilitation robotic hand that better realizes the recovery of hand function through the balance of FES and robot [73]. Researchers often stimulate FES with processed EMG signals or EEG that are produced from patients' spontaneous motor to contain spontaneous motor [74].

#### 3.3.6. Others

Other designs that corresponded with the active training modalities may not provide actuations, but totally actuated by patients' own hand; this means for a high requirement to patients' residual motor abilities [14]. Another compromised choice is the use of spring as actuation, in which the spring offers force to compensate the effect of hypermyotonia [75].

### 3.4. Transmission

The function of transmission is to transform the motion of actuator into a desired direction to complete the execution of a hand's motion. Most of these are a consequence of the choices of actuator or the mechanism.

#### 3.4.1. Linkage

Linkages are popular choices in the hand rehabilitation robot system, the same as in the traditional mechanical design. The linkages are light, convenient, and can be easily controlled in a given trajectory. On the one hand, the problem of coincidence of the rotational axis can be settled by using the cross-joint structure. On the other hand, the complexity of device can be reduced according to the concept of fDOF by using the linkage structure [24]. For example, Fontana et al. designed the cross-joint exoskeleton that uses the virtual joints to avoid the misalignment (Figure 3, c-1) [76]; the mechanisms designed by Wege et al. adopt a linkage structure connecting the adjacent finger segments [74]; and Fiorilla et al. designed the 2-finger hand exoskeleton that adopts the concept of fDOF to simplify the structure (Figure 3, c-2) [77]. The redundancy can be eliminated by this structure while offers an easy way to control the movement.

#### 3.4.2. Cable

The cable is also frequently used as the transmission in the hand rehabilitation robots, including the pulley cable and Bowden cable. The pulley requires a continuous tension to maintain traction on the pulleys, which limits the use [78, 79]. On the other hand, the Bowden cable is better for its cable conduit and is flexible. Disadvantages are the variable and high-friction force caused by the curve [74, 80]. For example, the cable actuated finger exoskeleton (CAFE) (Figure 3, d-1) designed by Jones et al. and the HandCARE (Figure 3, a-2) designed by Dovat et al. adopt the pulley cable [55, 78]. These devices can be easily controlled by force while not so convenient in usage. The robot designed by Wege et al. (Figure 3, d-2) adopts the Bowden cable to control the motion of fingers independently [19, 20, 74]. In fact, this robot combined both the cable and the linkage structure. Cables are similar to the muscle of the hand, so it might be an effective tool for the hand rehabilitation robotic system [81]. The jointless exoskeleton designed by In et al. is a good application of this concept [38].

### 3.5. Sensor

**Figure 4:**
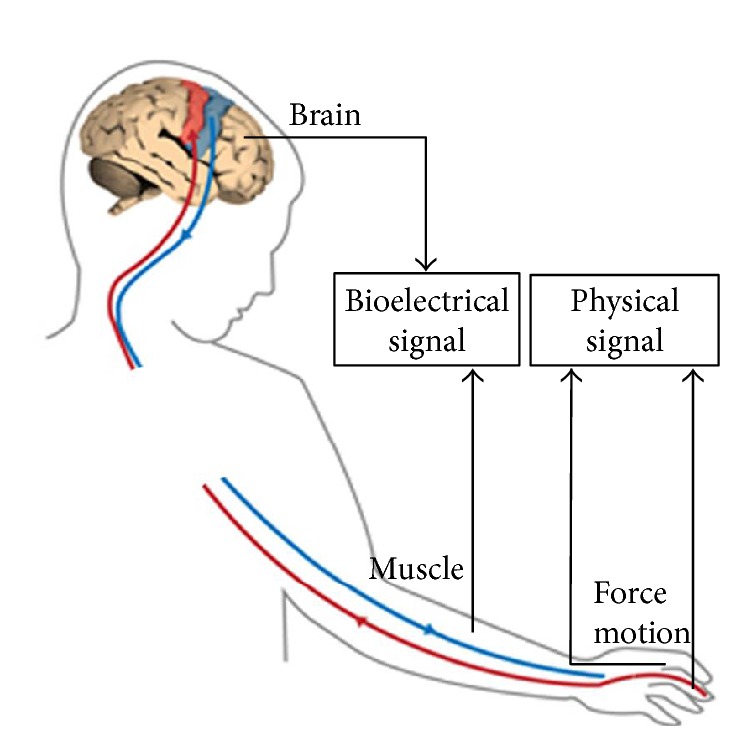
Sources of two kinds of signals.

Although the hand rehabilitation robot system has some effects just according to the continuous passive motion (CMP) concept, the participation of patients seems to be more effective in the rehabilitation system [82, 83]. This makes the sensor very important in the hand rehabilitation robot system. The sensor detects information of human to offer feedbacks or control signals to the human or robots. Here, the sensors are classified by the types of detected signals (Figure 4).

#### 3.5.1. Physical Signal

The sensors detecting physical signal such as the force and position (or motion) are the most used sensors in the robot system of hand rehabilitation [24]. The function of force or position signal is to provide the physical state of the hand such as the exerted force of motion or the bending angle of the finger [84–88]. For example, the sensing and force-feedback exoskeleton (SAFE) robotics was designed by Ben-Tzvi et al., in which an optical position sensor and strain gauges are set to detect the motion and force signal [89].

#### 3.5.2. Bioelectrical Signal

The other kinds of sensors detecting the bioelectrical signals such as the EEG or EMG signals are also frequently used in the hand rehabilitation robot system [24, 41]. The function of the bioelectrical signal is to reflect the motion intention of human, which can be used as the controlling signals of robots. The EEG and EMG signals are the most representative signal obtained from the brain or muscle, since other possible but inconvenient signals such as the magnetic resonance imaging (MRI) signal are not listed here [90]. Examples are SAFE that uses the EEG cap to detect the EEG signal from the brain and the robot designed by Hu et al. that uses several EMG electrodes arranged on the extensor digitorum muscle and abductor pollicis brevis muscle [40, 41].

## 4. Training Paradigm

The training paradigms designed according to the rehabilitation theories or clinical results with good efficacy might be the most crucial things in the motor recovery. The essence of training paradigms is how the robots interact with the patients from signal perception to physical contact. To date, no comprehensive reviews discussing the training paradigms are found, although some classifications such as training modalities and training strategies have been widely used in researches [14, 23]. For designing better application of rehabilitation robot system, the training paradigms are classified according to several aspects. These aspects are either already widely used such as the training modalities or being discussed more or less in the past but summarized in the authors' view such as the human-robot interaction.

### 4.1. Training Modality

The classification of training modalities relates to conventional therapy modes used in a clinical practice (Table 1) [14]. It refers to a subject's status during interaction and the properties of force applied to the hand. For example, the active or passive modalities reflect weather there is participation of the patients' intention in the motion. The assistive or resistive modality reflects the robot offer, the assistant or resistive force, for the motion of the hands. Other training modalities proposed by Basteris et al. are not classified here for they can also be classified under this classification [14]. For example, the passive-mirrored training modality belongs to the active-assistive modality in which the contralateral hand plays the role of the robot [14]; the path guidance training modality belongs to the assistive modality in which the assistant force of the robot turns to be a force field around a predefined trajectory.

#### 4.1.1. Resistive

In resistive training modality, the patient completes the motion under the resistive force offered by the robot. This kind of training mainly focuses on the force training, so the robot usually adopts the impedance control schemes [91, 92]. For example, Lambercy et al. designed the haptic knob for hand rehabilitation. The robotic system consists of two control schemes: in the opening course, the robot is controlled by the PID position controller and in the closing course, the robot offers a resistive force that composed of a constant force and a damping component [93, 94]. The resistive force is adapted to the subject's impairment level, and a significant homogeneous improvement of hand function was observed. Researches on the resistive training of the hand are relatively rare, most of which focus on other arm segments such as the shoulder, elbow, or wrist [95, 96]. The limitation of resistive training modality is that it requires the patient's residual motor ability being strong enough to overcome the resistive force of the robot. Therefore, it is better to be used for patients in the later period of stroke [14].

#### 4.1.2. Active

In active training modality, the patient executes the motion on his own ability, while the function of robots is being used as a measurement device to offer feedback to the patients. In this kind of training modality, the motion is totally controlled by human, while no control scheme is needed here. For example, Adamovich et al. studied the rehabilitation system in which the patients wore CyberGloves and CyberGrasp device to get the position and force information. When the patients execute motion on their own, the real-time information of the hand are collected and presented with a virtual scene and feedback force [97, 98]. In fact, researches on pure active training of hand rehabilitation are hardly observed, because most of these robots can be replaced by data gloves [99]. Besides, the active training requires that the patients have enough ability for independent motion [14, 100].

#### 4.1.3. Assistive

In assistive training modality, the patient executes voluntary motion of his hand with the assist of the robots. The robot offers continuous force to the hand during the motion. This kind of training modality can be as simple as the HandSOME designed by Brokaw et al., in which the assistant force is offered by a spring to counteract the muscular tension [75]. No control scheme is needed in this design, but the assistant force offered by the spring is difficult to adjust and may lack individuality for different patients. For assistive training, the training according to the motion predefined trajectory is usual. For example, the hand rehabilitation system designed by Wege et al. adopted the standard PID controller and the sliding control model [74, 101]. The PID controller gave satisfied results of position control, while the sliding control model is more robust and the trajectories are followed with good accuracy. Nevertheless, the assistive training modality requires that the patients have residual motor ability [102].

#### 4.1.4. Active-Assistive

In active-assistive (also known as active-passive) training modality, the patient completes the motion with the help of robots, but here, the robot would not offer force to the hand until the patient cannot move on its own. The active-assistive training modality is the most widely used one in the rehabilitation robot system [103, 104]. Active-assistive training can also adopt simple control scheme as the AMADEO did, in which the assistant force is only supplied when the patient could not complete the full range of motion [105]. These kinds of control scheme may not be so useful for patients with weak motion ability. More and more active-assistive training modalities adopt the biosignal as the input of control, but most of the current researches are based on the binary (on-off) control scheme, which limits the use of biosignal input. The muscle model and the fuzzy control are a new trend of control scheme with an advantage in active-assistive training modalities [106, 107], while more application on hand rehabilitation is needed. The assistive training modality requires the residual motor ability or at least that the patient can generate enough motor intention.

#### 4.1.5. Passive

In passive training modality, the motion of the patient's hand totally depends on the force offered by the robots. According to the continuous passive motion (CPM) concept, the massive passive training would promote the motor recovery. Robots of passive training modality are usually controlled by a position to do repetitive training, in which the robots move the hand from the start position to the end position and then move back [75, 108]. There are not much requirements to the interaction of humans and robots, thus most robots of other training modalities can train the patients in passive training modality [91, 105]. Although the passive training has been widely used in the rehabilitation robotic system for which requires no motion ability of the patient, it still has limitation for the participation of patients and is very important in the rehabilitation [91, 108].

## 5. Patterns of Movement

**Figure 5:**
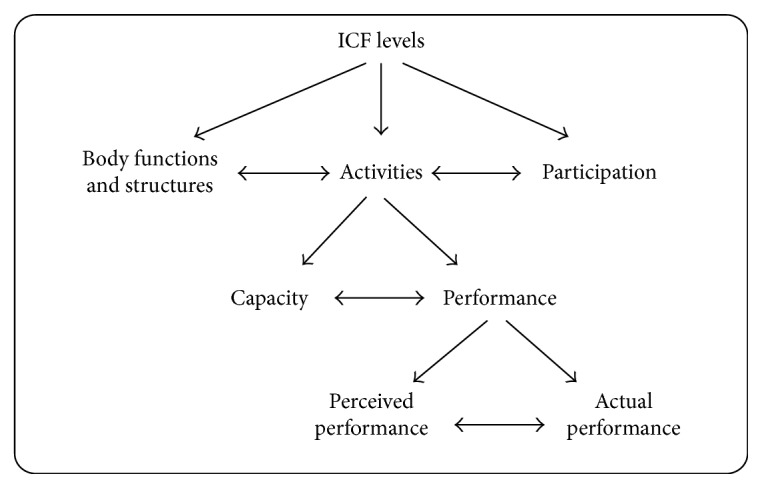
ICF levels and subdivision [109].

According to the collected papers, current design of movements in hand rehabilitation robots can be divided into two patterns. The first pattern focuses on the range of motion, muscle strength, and spasticity while the second one focuses on performance of functional tasks, especially for the ADLs. This is corresponding to the International Classification of Functioning, Disability and Health (ICF) framework (Figure 5), in which the human functioning is described at three levels, that is, functional level (body structures and function), activity level (task execution), and the participation level (involvement in life situations) [109, 110]. Although initial results of meta-analyses on clinical trial results with robotics showed the effectiveness of robotics for improvement on functional level, no improvement on the ICF activity level is demonstrated. It might be caused by people's poor understanding of the relationship between function level and activity level, but now, the focus of rehabilitation on hand disorders is slowly shifting from ICF function level towards activity and participation levels [35, 109–113].

### 5.1. Movement on Functional Level

Most of current design adopts the movement on functional level, that is, movements that focus on increasing the joint range of motion, improving muscle strength, and decreasing spasticity and so on. Intensively executing simple motion on one or several joints by such as continuously grasp or extension of palm is usually used in this kind of training [111, 112]. Movement pattern on functional level is a key point in rehabilitation for it decides the foundation of normal movements [109].

### 5.2. Movement on Activity Level

Movement on activity level can usually be found in task-specific training or training of activities of daily living (ADL) [31, 36, 114–116]. The movement in activity level is not as rigid as in the functional level, for example, the training with the soft robotic glove designed by Polygerinos et al. is to grasp blocks in a box [60]. The activity level is directly related to the performance of patients' abilities in actual life.

## 6. Human-Robot Interaction

The differentiation between different training paradigms is essentially decided by different human-robot interactions which are also called training strategies in other papers. Human-robot interaction is essential for the motor recovery, because in rehabilitation robotic system, the subject of human is the key point, which is often inadequately considered. This is different from traditional industrial or field robots that execute motion automatically or with the explicit command from humans, because the humans are both the commander and a component of the robot system of hand rehabilitation [26]. For human-robot interaction, the induction, intention, and feedback to the brain are the three main components. The induction induces motor intention for motion; the robots detect the right intention and then give augmented or transformed feedback to the patient. These components form a closed neuronal pathway for a motor.

### 6.1. Patterns of Induction

A number of innovations combined methods such as action observation or motor imagery with rehabilitation robotics. These different combinations could be generalized according to the different patterns of inductions. The induction is used to induce motor intention from the patient. It seems that the better induction pattern should be able to induce stronger motor intention and inspire more motivation of the motion from patients [39, 40]. There are three effective induction patterns, namely, the task-specific induction, mirroring induction, and virtual induction. Patterns such as explicit visual or auditory cue for the execution of motion are not described here for the weak effects in inducing intention. Nevertheless, these inductions can be presented before the execution of motion or be interactive with the results of motion execution.

#### 6.1.1. Task-Specific Induction

The task-specific (or task-oriented) induction usually offers real-life objects with which the patients perform motion of daily activities such as grabbing a bottle [117] or picking up blocks [60]. It is performed to be therapeutic interventions in almost all studies for the task-specific training, while others show hardly any generalization to the improvement on the ICF activity level [109, 118]. The intention induced by the task-specific induction is oriented to daily tasks, which is effective in improving activities in daily living for compensating the absence of activity-related training input in other therapies [119].

#### 6.1.2. Mirror Induction

The mirror induction refers to the mirror therapy or bilateral training (i.e., mirror training). In traditional mirror therapy, by using a mirror that is positioned orthogonally in front of the patient, the reflection of the right arm in the mirror provides an illusion that the left arm is being moved [120]. The mirror therapy has been used for many years and demonstrated to be useful in motor recovery [120, 121]. The bilateral training, in which the patients execute mirrored motion of both hands with the help of robots, is very similar to the mirror therapy [39, 40, 122]. The only difference is that the mirror illusion is replaced by the real motion of paretic hand under the assist of robots. The motor intension of paretic hand induced by mirror induction might be explained by the function of intercallosal fibers or the mirror neuron system (MNS) [123]. The combination of robots with bilateral training has been widely used while the combination with traditional mirror therapy has not been found.

#### 6.1.3. Virtual Induction

The virtual induction refers to virtual objects presented by computers [124]. There are three main thoughts to use virtual induction. First, using computers to realize the virtual task-specific induction, this has been demonstrated to be as effective as the task-specific training with the help of therapists [124]. Second, using videos of others' motion to induce motor intention according to the MNS has been more and more applicable [122, 125]. Third, using interesting game paradigm to induce stronger motor intention can be an induction mixed with the former two and adds to the motivation of executing the motion [126, 127].

The efficiency of game paradigm is promoted by findings that show immersive training, or implicit practice can strengthen the learning ability of patients [128, 129]. It should also be mentioned that the development of the VR technology makes the virtual more promising in hand rehabilitation robot system [98, 130, 131].

### 6.2. Detectable Intention

In a normal pathway of healthy subjects, the motor intention can be conducted to the muscle in electrical signal to actuate motion of the hand directly, while in a pathway of stroke subjects, the motor intention has to be detected by the robots and then the robots actuate the motion of the hand. The intention can be detected from the brain, muscle, and both the paretic hand and the contralateral healthy hand.

#### 6.2.1. From Brain

The EEG signals are represented brain signals, which contain the motor intention that can be detected. The EEG signal is the most convenient signal in the brain-computer interface (BCI) system. The BCI technology has been greatly developed in decades and has many effective applications in the robot system of hand rehabilitation. The application of BCI technology is mainly based on the fact that the motor imaginary (MI) promotes the recovery of limbs. The effectiveness of MI in rehabilitation has been demonstrated long before. Researches show that through the MI of limb movements, the feeling of paretic limb would be aroused to some extent for the motor cortex would be activated during MI, though the patients did no relevant motion [132–137]. Combined with the BCI technology, the signal of brain evoked by the patients' spontaneous MI can be extracted and classified to control the rehabilitation robotics directly. With the participation of MI in the brain and movement of the hand, the rehabilitation robotics realizes an artificial pathway to replace the normal motor pathway of humans and has a wide future [138].

Biebaumer is the earliest one to demonstrate that stroke patients can evoke MI-related ERD/ERS as the health individuals [139, 140]. Unexpectedly, researchers find that the muscular tension of these stroke patients reduced after participating in the experiment. Different research teams made further research based on this. Ang et al. conducted many experiments and verified that there are great possibilities for stroke patients to evoke the MI-BCI, thus making the application of MI-BCI possible [141, 142]. Barsotti et al. designed a full upper limb robotic exoskeleton by MI-BCI. After evoking the MI-BCI, the patients can achieve the motion of reaching and grasping [21, 22]. It can be found from these examples that the advantage of intention detected from the brain is promising in hand rehabilitation robots, because it makes the direct training of brain possible. But, the weaknesses of the current studies are that the real-time problem was caused by processing of large EEG data and that the accuracy of recognition rate of correct motor intention limited the effect of actual application [83].

#### 6.2.2. From Muscle

The electromyography signal of stroke patients is not enough to drive the motion of paretic hand, but it is strong enough for the data collection instruments to collect, thus it provides a new method of control for rehabilitation robotic hands, which is to control the motion of robotic hand with processed signals collected from the muscle. Researchers like Ho et al. designed electromyography- (EMG-) driven hand robots [143–145]. After the patient spontaneously produces a motor intention, the device collects and filters EMG signals, then controls the motor of the robotic hand with the processed signals. The experiment was conducted on 8 chronic patients, and the result verified the effectiveness of the rehabilitation robotic hand in the recovery of hand functions because the FMA scores of patients were improved. Besides, Fleischer et al. designed the EMG-driven rehabilitation robotics combined with force sensors for the legs or hands. This research combined with EMG improves the control strategy that purely uses the force signal [20]. However, that research was only developed for the legs, and no relevant research on hand had been found yet.

The EMG technology detects motor intentions directly from the EMG signal of patients, to help the execution of initiative motion. That is why it has better effects than the traditional passive training without the participation of muscles. However, there are still some points that should be focused for EMG-driven rehabilitation robotics. First, the EMG signal varies in the different conditions of stroke patients, so the rehabilitation robotic system should be adaptive [146]. Second, the current EMG devices should be able to recognize more types of hand motion, to achieve better effectiveness in recovery [147]. Kiguchi developed an EMG rehabilitation robotic device based on the fuzzy control that realizes the recognition of many types of motions of wrist and forearm. However, no relevant research refers to recognition of motions of fingers [107].

#### 6.2.3. Force and Motion on Hand

One of the most direct methods of sensing the user's intention is to measure the force exerted by the patient at the interface [25]. This method has been applied to many hand exoskeletons for assistance applications [89, 148–150]. Similarly, the initial movement pattern of the user's finger can also be a triggering command for programmed grasping based on a pattern classification technique [151]. The advantage of intention detected from force and motion on hand is that more information can be obtained during all the motion courses, compared with the EEG or EMG signal that contains too less information to be a variable control input [22, 143, 145]. The limitation of force and motion on the hand is the requirement of residual motion to patients; in other words, severe myasthenia patients may not be able to use these robots well [25, 83].

### 6.3. User Feedback

Although the human has its own proprioception, it is either abnormal or too weak for stroke patients. The robot can offer extra feedback to the brain to form an integrated close-loop pathway, which is meaningful for the human-robot interaction. The feedback is usually an augmented compensation for an attenuation of haptic feedback [152, 153]. It can be visual, auditory, haptic, or multimodal. The feedback can be divided as two types: feedback of motion state and feedback of motor performance.

#### 6.3.1. Feedback of Real-Time State

The purpose of using the feedback of real-time state is to compensate the absent self-feedbacks such as haptic feedback of the hand. The feedback can be the visualization of force exerted to the device, index of EMG/EMG signals, or joint motion compensator realized by actual mechanism [152, 154–156].

#### 6.3.2. Feedback of Motor Performance

The feedback of motor performance is mainly used in a game paradigm. The efficiency of this feedback might be explained by the simple Pavlovian conditioning. The award of completing a motion presented by a computer program would reinforce the motor feedback loop and inspire more motivations in the next motion [157]. Nevertheless, the somatosensory feedback of completing an actual movement is also an effective feedback that helps direct brain reorganization [40].

## 7. Discussion

As the classification shows, varied kinds of design of hand rehabilitation robots have been explored in all these studies, thus making the hand rehabilitation robotics being greatly developed in recent years. However, the progression of clinical use of hand rehabilitation robots has not caught up with the pace of the designing of the prototype. The result of an evidenced-based analysis even shows that many other alternative interventions have “stronger evidence” than the robot-assisted therapy [50]. Here, the general limitations of current researches on hand rehabilitation robots are given to remind future studies. Experience and prediction in the research of both hardware system and training modalities are discussed.

## 8. General Limitation

One obvious general limitation of current researches is that many researches ignore the importance of evaluating the design of hand rehabilitation robots, which could be found from Table 2. There are mainly three kinds of ways to deal with the evaluation of designs in collected studies. The majority of studies adopt the first way that gives no evaluation of robots, making no effects on promoting the practical use. This makes the design unconvinced for patients or therapists to adopt it although the robots might seem to be ingenious. A number of studies adopt the second way of evaluating designs by giving few tests on the improvement of robots' physical parameters or of the performance on specific scenes. Physical parameters varied like the ROM of joints, the velocity of motion, and the assistant force exerting to hands, while the specific scenes that like the accuracy of reaching a target are totally set up by the investigator subjectively. This kind of evaluation differs from each other, thus making it difficult to tell which design is more available than the other one [39, 40, 75, 164]. Few studies adopt the last way that gives the evaluation of the efficacy of designs combining in the clinical methods such as the improvement of clinical scale which is the most acceptable method in rehabilitation. Although many independent researches show abundant successful design, a systematic research under the principle of EBM gives weak demonstration for the usefulness of rehabilitation robots [45]. This could be explained that most studies suffered from methodological shortcomings, such as a lack of blinding procedures and intention-to-treat analysis, which may have resulted in a positive bias in reported effects.

A solution is that more standard randomized controlled trials (RCTs) should be designed on evaluating current robot system of hand rehabilitation. On the one hand, the approach of using clinical scale which is easy to administer for assessment should be popularized in hand rehabilitation robotic design, for it is the most acceptable way in clinical use now. On the other hand, to set other standardized evaluation methods realized by more objectives with the help of robots might be promising, for it overcome the biggest disadvantage of clinical scale of being subjective for the visual scores of the test [30, 170, 171]. The measurement robots can benefit both the evaluation of robot design and the assessment of rehabilitation degree [170, 171].

Lacking of pertinence is another limitation of the current design. It can be found from Table 2 that only few studies give specific discrimination of subjects, while the motor impairment of these patients nearly varies from person to person for the complexity of stroke sequela [54, 75, 169]. The different factors such as phases of stroke and muscular tone may have very different requirements to the hand rehabilitation robots. For example, acute patients experiencing muscular weakness may not be able to trigger the robot controlled by force signal; muscular hypertonia might gain abnormal motion patterns if their grasp strength is overtrained by robots. Executing repetitively programmed motion is the advantage of robots, while the individualized motion for specific patient is meaningful. By the way, clinical experience from traditional therapy such as the division of recovery stages in the Brunnstrom approach can also be used for references for pertinence designs [151]. All these factors are crucial for the rehabilitation in deciding the intensity of training and the proper training modalities in a customized design.

### 8.1. Hardware System

The function of a specific HRR can often be realized in several different combinations of different hardwares. What decides the efficacy of HRR is not a single hardware but the whole hardware system that makes choosing of hardware not just finding an absolutely best hardware but trading off different components under a specific need of application.

The exoskeleton robots are the dominant choice compared with end effector robots under the trend of in-home treatment. Although the end effector has the advantage in omitting the problem of the coincidence of joints, this merit can also be realized by underactuated exoskeleton robots based on the notion of fDOF. Besides, a lot of expense can be reduced, because the controlled DOF can be decreased according to the fDOF notion. The promotion of in-home treatment makes the application of end effector robots fewer in hand rehabilitation because of constraints on portability.

Choosing of actuation is mainly between the electrical motor and the pneumatic artificial muscle. The merits that like being easy to control, with high precision and with high energy-to-weight, make the electrical motor the prior actuation in hand rehabilitation robot. Being portable and small in volume are very crucial for designing a flexible robotic hand. The development of soft-bodied robots makes the pneumatic actuation another noticeable choice in robotic hand. The robots using pneumatic artificial muscle can be small and with high safety compared with traditional actuations, while the application of soft-bodied robots for hand rehabilitation is still limited by the extra gasholder.

Among the choice of actuations, the contralateral extremity and the human muscle should be more focused on. The mirror therapy, although with undefined mechanism, has already gotten good performance under many practical uses in conventional therapy and several initiatory applications in robot-assisted therapy [165, 166]. It should be mentioned that current studies on hand rehabilitation robots realize the mirror therapy in an indirect method through conventional actuators controlled by signal from data glove, while studies using direct mechanical method are not covered. The success of treatment combined with FES and EMG can also inspire the notion of combing robots and FES. Serea et al. have realized a system according to this notion [83, 172]. These two kinds of actuation are both promising for the interaction between the human hand and robots, thus demanding a higher level of choosing sensors. Further studies on exploring the better new ways of processing information from hands and robots, instead of just being a trigger of motion, are also demanded for robots with better efficacy of rehabilitation.

Apart from soft body structure, the linkages are almost the necessary among the transmissions. Using the linkages to form an underactuated robot is the mainstream method in solving the problem of joint coincidence. Although the soft body structure has the advantage in being adaptive to human bodies, the complexity on control system and the problem of portability limit its application. It should be mentioned that the jointless robots designed by In et al. using the cable provide new thoughts in using cable to solve the motion problem [38].

The extensive use of sensors for bioelectrical signal is the tendency in design of hand rehabilitation robot. Although the sensor is not necessary for a robotic hand, the consensus on the importance of patient's participation demands sensors for detecting signal from human bodies. The bioelectrical signals that were obtained directly from the delicate neural activities expend the methods of detecting signal form the patients. Even though the patients cannot move their hands, the bioelectrical signal can also be used in place of the physical signal. Endeavor on exploring the sensors for bioelectrical signal should also focus on solving the problem of lacking of effective features for control signals, because the motion of the hand is varied.

### 8.2. Training Paradigm

Choosing of training paradigms decides the kernel of a hand rehabilitation robot, while it has not been fully introduced in papers combined with the whole rehabilitation robot system in consideration of patient difference and the merit and demerit of different designs.

Choosing of training modalities should better be nonconstant in the whole period of training for maximizing the efficiency of hand rehabilitation robots, in correspondence with the practical condition of patients. Two demerits for current choosing of training modalities: First, in passive motion, the absence of patients' active participation slows down the process of motor relearning; while, second, in positive motion, most patients in early stage are too weak on both muscle strength and mental activity, thus making the training process hardly inaccessible for them. Adopting varied modalities as the state changes in the rehabilitation may make a difference. For example, use passive training in the early stage of treatment for patients with difficulty in generating detectable intention to restore the muscular tone; use active-assistive training in the midstage of treatment to promote the process of motor relearning; and use active training in the later stage of treatment for patients to regain independent motor ability in case of overreliance. Besides, the control scheme is better adopted according to different training modalities. For example, for the resistive training modality, the impedance control is a good choice, because the impedance control can allow more dynamic interaction with the patients. Fuzzy controlled may be a good choice for active-assistive training, because the participation of intention detected from biosignal can be better classified in this control scheme [107, 173]. It should be mentioned that choosing of right modalities still needs many clinical data for making better decision according to more sufficient research support.

Movement on activity level would be the focus in subsequent studies. It could be found in Table 2 that many studies such as the hand rehabilitation robots controlled by EMG signals succeeded in restoring the human function on function level, but then stopped before the further rehabilitation [41, 118, 145]. The fact is that the restoring of function on an activity level is more meaningful to patients for it decides the independent ability in a daily life. The hand rehabilitation robots with goal-oriented design seem to be in a very good direction of rehabilitation [174]. With the help of computer science, many ADL can also be realized in a virtual world. Some research shows that somatosensory feedback through rehabilitation robots is more effective at improving finger motor function than animated visual feedback on a computer screen [84].

**Figure 6:**
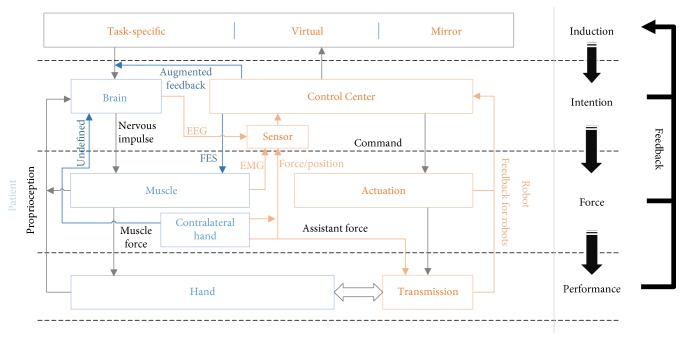
The relationship between the human motor system and the hand rehabilitation robot system.

Human-robot interaction should be taken more into consideration for it decides the basic mode of the robot's role in motor recovery of patients' brain, and it connects the hardware system with the training paradigms. This is provided in Figure 6, from which the comparison of the rehabilitation robot system with the human motor control system is shown. The basic method of designing hand rehabilitation robot can be regarded as designing an artificial system to make up the incomplete human system and promotes the relearning process of human system. The hand rehabilitation robot has been developed form the earliest passive robots to robots with feedback and controlled through information of muscle or brain information. Although the hand rehabilitation robots are developed more like the human, there is still distance between the real human system and hand rehabilitation robot system.

First, the system in most designs is not real-time but has been provided through long time-window sequence. This makes the hand rehabilitation robot system a not real closing loop system and separates the perception of execution and feedback of patients [175, 176]. For example, in the detection of human intention, the time-delay problem is caused by processing the vast and complex data of EEG signal. This needs more effort on finding the better classification algorithm, such as the research by Gomez-Rodriguez et al. that uses a time window to detect a real-time intention. The time-delay problem also exists in the feedback of real-time motion information. Efforts on designing better sensing circuit of hardware are needed to solve the problem.

Second, the humans have varied intentions while current researches could only figure little of them. The more closing the intention is detected from the central nervous system, the littler intention could be detected, while more intention can be detected from the distant extremity. Besides, current technologies on detection intention from the brain or muscle are either low in accuracy or inconvenient in clinical use. On the one hand, better detection system should be developed. On the other hand, although motions of distant extremity are difficult to be motivated, they still give us a tip that the combination of different intentions might be excellent in both varieties and motivation [177].

## Figures and Tables

**Table 1:** Different demands in the hand rehabilitation robotics with different training modalities.

Training modalities	Patient		Robot
	Intention	Force	Force
Passive	**0**	**0**	**+++**
Assistive	**+**	**+**	**+**
Active-assistive	**++**	**+/0**	**0/+**
Active	**++**	**+**	**0**
Resistive	**++**	**++**	**—**

For the force of robots/patients: + means the assist of force on motion; − means the more resist of force on motion; 0 means no assist or resist of force on motion. For the intention of patients: + means the participation of patient's voluntary intention on motion; 0 means no participation of patient's voluntary intention on motion. The bigger counts are corresponding to the higher requirement to each object.

**Table 2:** An overview of current designs of hand rehabilitation robots.

Device	Reference			Subject			Hardware system				Training paradigm						Evaluation				
	Study	Year	ID	Phase	Muscle status	Extent of stroke	Types of robots	Actuation	Transmission	Sensors	Training modalities	Movement patterns	Induction	Detected intention	Feedback	Experimental subject	ROM	Velocity	Force	Functional testing	Clinical scale
Hexosys I	Iqbal et al.	2010–2014	[158]	N/A	N/A	N/A	Exoskeleton	Electrical motor	Linkage	Force/position sensor	P/AA	N/A	N/A	N/A	—	—	—	—	—	—	—
Hexosys II	Iqbal et al.	2011	[159]	N/A	N/A	N/A	Exoskeleton	Electrical motor	Linkage	Force/position sensor	P/AA	N/A	N/A	N/A	—	—	—	—	—	—	—
Hexorr	Schabowsky et al.	2010	[160]	N/A	N/A	N/A	Exoskeleton	Electrical motor	Linkage	Torque sensor	AS	N/A	N/A	N/A	—	—	—	—	—	—
Handexos	Chiri et al.	2009	[53, 58]	N/A	N/A	N/A	Exoskeleton	Electrical motor	Cable, crank-slide	—	P/A	N/A	N/A	N/A	—	—	—	—	—	—	—
Handsome	Brokaw et al.	2011	[75]	N/A	Hypertonia	Severe to moderate	Exoskeleton	Spring	Linkage	Position sensor	AS	Functional level and activity level	N/A	N/A	—	8 chronic patients	MAX ROM + 48.7° (*P* < 0.001)	Peak velocity + 67°/s (*P* = 0.004) Velocity of extension + 48°/s (*P* = 0.053)	Grip strength −3.7 N (*P* = 0.17)	Lifting larger block (*P* = 0.02)	—
Hand-assist robot	Ueki et al.	2004–2014	[39, 159–161]	Acute	N/A	N/A	Exoskeleton	Electrical motor/contralateral extremity	Linkage	Torque/force sensor	AA	Functional level	Mirrored motion VR	Joint angles of unaffected hand	Real-time state by VR	Healthy subjects	N/A	N/A	N/A	Self-control experiment	—
Sarakglou et al.	Sarakglou et al.	2004	[40]	N/A	N/A	N/A	Exoskeleton	Electrical motor	Linkage pull cables	Position/force sensor	AA	Activity level	VR	Force on hand	Real-time state by VR	—	—	—	—	Virtual tasks	—
Fingerbot	Cruz E G	2010	[164]	Chronic	Hemiparesis	N/A	End-effector	Spring/electrical motor	Linkage	Position/force	AS/A	—	—	—	—	10 chronic patients	Larger active ROM	—	—	Greater accuracy in terms of reaching the target	—
Ramos	Ramos	2009–2012	[42]	Chronic	N/A	N/A	End-effector	Electrical motor	Cable	Position/force	P/AA/A	N/A	—	Intention in the brain	—	23 health subjects	—	—	—	—	—
Mricr	Tang et al.	2011–2013	[165–168]	N/A	N/A	N/A	Exoskeleton	Ultrasonic motor	Linkage	Position	P/AA/A	Functional	—	Intention in the brain	—	—	—	—	—	—	—
AMADEO	Pinter D	2010–2014	[54, 169]	Acute/chronic/mixed	N/A	Moderate-to-high grade	End-effector	N/A	N/A	Position	P/AS/AA/A	Functional	VR	Position	Real-time state by VR	Multiple	—	—	—	Improved hand function	FM scores improved

N/A: the content is not available; —: the design in the content is null; P: passive; A: active; AS: assistive; AA: active assistive; R: resistive.
